# Ni-Modified Defect-Engineered NH_2_-UiO-66 for Efficient H_2_O_2_ Photosynthesis Coupled with Benzyl Alcohol Oxidation

**DOI:** 10.3390/nano16100626

**Published:** 2026-05-19

**Authors:** Yuan Chang, Zhenzi Li, Xuepeng Wang, Shuhua Liu, Bo Wang, Lijun Liao, Wei Zhou

**Affiliations:** 1 School of Chemistry and Chemical Engineering, Qilu University of Technology (Shandong Academy of Sciences), Jinan 250353, China; tanran2024@hotmail.com (Y.C.); zzli@qlu.edu.cn (Z.L.); wangxuepeng@qlu.edu.cn (X.W.); wangb123@qlu.edu.cn (B.W.); 2 Shandong Analysis and Test Center, Qilu University of Technology (Shandong Academy of Sciences), Jinan 250353, China; 3 School of Chemical and Blasting Engineering, Anhui University of Science and Technology, Huainan 232001, China; lijun.liao@aust.edu.cn

**Keywords:** photocatalysis, hydrogen peroxide, NH_2_-UiO-66, Ni modification, defect engineering

## Abstract

Photocatalytic H_2_O_2_ production coupled with selective organic oxidation provides a promising strategy for simultaneously generating value-added oxidants and chemicals under mild conditions. Herein, Ni-modified defect-engineered NH_2_-UiO-66 photocatalysts (Ni/UN) are constructed by introducing Ni species into a vacuum-treated NH_2_-UiO-66 framework (UN). Compared with the original NH_2_-UiO-66 and the defect-treated UN, Ni/UN exhibits weakened photoluminescence emission, enhanced transient photocurrent response, and reduced electrochemical impedance, indicating that the separation and transfer of photogenerated charge carriers have been improved. The band structure analysis further reveals that Ni/UN has a narrow band gap of approximately 2.52 electron volts and a slightly more negative conduction band position (−0.50 V), which is conducive to the photoinduced reduction reaction. The importance of O_2_ in the photocatalytic process was demonstrated by changing the atmospheric conditions. Therefore, in the benzylalcohol system, under the oxygen atmosphere, Ni/UN achieved the highest H_2_O_2_ production rate of 3257 μmol g^−1^ h^−1^, accompanied by the continuous generation of benzaldehyde, with its content reaching 3420 μmol g^−1^ after 60 min of irradiation. The scavenger experiment further indicates that photogenerated electrons and the active substances derived from oxygen are closely involved in the formation of H_2_O_2_, while the ·OH-related processes only play a limited contribution role. This study demonstrates an effective strategy for enhancing the performance of metal–organic framework (MOF)-based photocatalysts through defect engineering and metal coordination regulation, thereby achieving efficient photochemical production of hydrogen peroxide and the selective oxidation of benzyl alcohol.

## 1. Introduction

Hydrogen peroxide (H_2_O_2_) is an environmentally benign and value-added oxidant that has been widely used in disinfection, wastewater treatment, bleaching, chemical synthesis and emerging energy-related processes [1,2,3]. At present, the industrial production of H_2_O_2_ is mainly dominated by the anthraquinone process, which generally requires multistep hydrogenation and oxidation cycles, noble-metal catalysts, organic solvents, centralized production facilities and energy-intensive separation procedures [4,5]. These limitations have stimulated increasing interest in developing decentralized and sustainable routes for H_2_O_2_ synthesis under mild conditions [6]. Among various strategies, solar-driven photocatalytic H_2_O_2_ production from molecular oxygen has attracted considerable attention because it enables the direct conversion of light energy into chemical oxidants through interfacial redox reactions [6,7]. However, the practical efficiency of photocatalytic H_2_O_2_ synthesis is still restricted by rapid recombination of photogenerated charge carriers, insufficient activation of O_2_, sluggish surface reaction kinetics and competitive side reactions, including the over-reduction in O_2_ or the decomposition of generated H_2_O_2_ [4,8,9]. Therefore, rational construction of photocatalysts with enhanced charge separation ability and optimized O_2_ activation sites remains essential for improving H_2_O_2_ photosynthesis [10,11].

In addition to photocatalytic H_2_O_2_ generation from water and oxygen, coupling O_2_ reduction with selective organic oxidation provides a promising route to improve the utilization of photogenerated holes while simultaneously producing value-added oxidation products [3,11,12]. Benzyl alcohol is a representative substrate for this purpose because its oxidation product, benzaldehyde, is an important intermediate in fine chemical synthesis [3,13]. In such a reaction system, photogenerated electrons can participate in O_2_ reduction to produce H_2_O_2_, while photogenerated holes are consumed by benzyl alcohol oxidation, thereby alleviating charge recombination and avoiding unproductive oxidation processes [11,14]. Compared with conventional sacrificial-agent systems, the benzyl alcohol system is more attractive because the oxidation half-reaction is directly linked to the formation of a valuable product rather than simple hole consumption [13,14]. Nevertheless, efficient H_2_O_2_ production in this system requires photocatalysts that can simultaneously promote visible-light harvesting, charge migration, interfacial O_2_ activation and selective benzyl alcohol oxidation [15].

Metal–organic frameworks (MOFs) have emerged as promising photocatalytic platforms owing to their crystalline porosity, tunable metal nodes, designable organic linkers and adjustable coordination microenvironments. Among them, amino-functionalized [16,17] UiO-type Zr-MOFs are particularly attractive because the aminoterephthalate linker can extend visible-light absorption, while the robust Zr–oxo cluster provides relatively high structural stability under photocatalytic conditions [18,19]. More importantly, defect engineering in UiO-type frameworks can regulate the local coordination environment, expose unsaturated sites and provide anchoring positions for guest metal species, thereby influencing charge transfer and surface catalytic reactions [20,21]. However, pristine NH_2_-UiO-66 still suffers from limited charge separation efficiency and insufficient interfacial catalytic activity, which restricts its application in efficient photocatalytic H_2_O_2_ synthesis [22]. Therefore, integrating defect modulation with metal-site regulation represents a feasible strategy for constructing NH_2_-UiO-66-based photocatalysts with improved redox performance [22,23,24].

Transition-metal modification has been demonstrated to be an effective approach for tuning the electronic structure and surface reaction behavior of photocatalysts [25,26,27]. In particular, Ni-based sites have attracted attention because they can participate in the adsorption and activation of oxygen-related intermediates, thereby influencing the oxygen reduction pathway toward H_2_O_2_ formation [22,28]. Previous studies have shown that Ni single-atom sites in carbon nitride could facilitate the formation of *OOH-related intermediates and promote photocatalytic H_2_O_2_ production [10,28]. Similarly, Ni single atoms immobilized in defective Hf-UiO-66-NH_2_ were reported to enhance H_2_O_2_ photosynthesis from O_2_ and H_2_O, suggesting that the combination of framework defects and Ni coordination sites could be beneficial for constructing efficient MOF-based photocatalytic systems [28]. Despite these advances, the role of Ni incorporation in defect-engineered NH_2_-UiO-66, especially in a benzyl alcohol system for H_2_O_2_ generation coupled with benzyl alcohol oxidation, still requires further investigation.

Herein, Ni-modified defect-engineered NH_2_-UiO-66 photocatalysts, denoted as Ni/UN, were constructed by introducing Ni species onto a vacuum-treated UN. Structural characterizations demonstrate that the main UiO-type crystalline framework, characteristic vibrational features and octahedral morphology are retained after Ni loading, indicating that Ni incorporation does not induce obvious framework collapse. XPS analysis confirms the successful introduction of Ni species, with Ni mainly existing in the divalent state and possibly interacting with oxygen species associated with the Zr–oxo cluster. Compared with pristine NH_2_-UiO-66 and UN, Ni/UN exhibits weakened PL emission, enhanced transient photocurrent response and reduced electrochemical impedance, indicating improved separation and migration of photogenerated charge carriers. Meanwhile, band-structure analysis shows that Ni/UN possesses a narrowed band gap and a slightly more negative conduction-band position, which is favorable for photoinduced reduction reactions. Benefiting from the synergistic effect of defect-engineered NH_2_-UiO-66 and Ni modification, Ni/UN delivers the highest H_2_O_2_ production rate among the tested samples, reaching 3257 μmol g^−1^ h^−1^ under O_2_ atmosphere in the benzyl alcohol system. The atmosphere-dependent experiments reveal that H_2_O_2_ generation is markedly promoted under O_2_ compared with air and N_2_, confirming the crucial role of molecular oxygen in the reaction. Meanwhile, the continuous formation of benzaldehyde during irradiation demonstrates that benzyl alcohol oxidation occurs at the oxidation side, indicating that H_2_O_2_ production is coupled with selective organic oxidation. Scavenger experiments further show that photogenerated electrons and oxygen-derived reactive species are closely associated with H_2_O_2_ formation, whereas ·OH-related processes make only a limited contribution under the present conditions. This work provides a Ni-coordination-regulated defect NH_2_-UiO-66 photocatalyst for efficient H_2_O_2_ generation coupled with benzyl alcohol oxidation, offering insight into the design of MOF-based photocatalysts through local coordination environment modulation and charge-transfer optimization.

## 2. Results and Discussion

**Figure 1:**
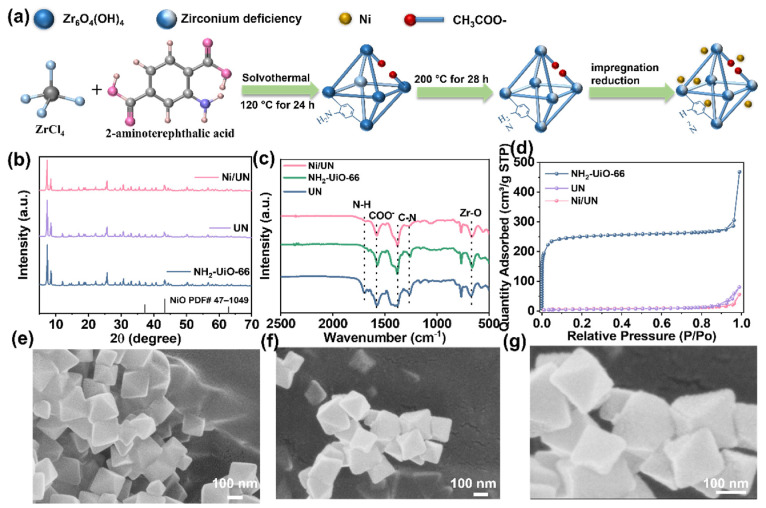
(**a**) Schematic illustration of the preparation route of Ni/UN. (**b**) XRD patterns of NH_2_-UiO-66, UN and Ni/UN. (**c**) FTIR spectra of UN and Ni/UN. (**d**) N_2_ adsorption–desorption isotherms of NH_2_-UiO-66, UN, and Ni/UN. (**e**–**g**) SEM images of (**e**) NH_2_-UiO-66, (**f**) UN and (**g**) Ni/UN.

As illustrated in Figure 1a, Ni/UN was prepared by introducing Ni species into the defective UN, aiming to construct a metal-modified NH_2_-UiO-66-based photocatalyst. The detailed synthesis method can be found in the Supporting Information. The XRD patterns in Figure 1b show that UN and Ni/UN retain the main diffraction features of NH_2_-UiO-66, indicating that neither the high-temperature vacuum treatment nor the subsequent Ni loading destroys the long-range ordered framework of the parent MOF [29]. Compared with the UN, the diffraction peak positions of Ni/UN remain essentially unchanged. In addition, no obvious diffraction peaks assignable to crystalline NiO are observed when compared with the NiO standard card PDF#47-1049, suggesting that no detectable large crystalline NiO phase is formed in the present samples. However, this result alone cannot exclude the presence of highly dispersed, low-content, or poorly crystalline Ni species. This interpretation is consistent with the general understanding that metal species introduced into MOF-based hosts at low loading can be difficult to identify by XRD when they are highly dispersed or weakly crystalline.

The FTIR spectra in Figure 1c further confirm the preservation of the NH_2_-UiO-66 framework after Ni incorporation. The characteristic band at around 1580 cm^−1^ can be assigned to the vibration of carboxylate groups [30], while the band near 1700 cm^−1^ is related to the N-H vibration [22]. The low-wavenumber signal around 660 cm^−1^ corresponds to the Zr-O vibration [31], and the bands at approximately 1380 and 1260 cm^−1^ are associated with the C-N vibration [30]. Compared with UN, Ni/UN shows no disappearance of the main framework-related vibrational bands, demonstrating that Ni loading does not significantly alter the basic MOF skeleton. Nevertheless, slight changes in peak intensity and shape can be observed, implying that the introduction of Ni may affect the local coordination environment around defect sites, hydroxyl groups, or carboxylate linkages rather than generating a new dominant framework phase.

The N_2_ adsorption–desorption isotherms in Figure 1d reveal the evolution of the pore structure after defect construction and Ni loading. NH_2_-UiO-66 exhibits a rapid adsorption increase at low relative pressure, reflecting its typical microporous character. After high-temperature vacuum treatment, UN shows a lower N_2_ uptake, indicating a decrease in accessible surface area. After further loading Ni, Ni/UN displays an additional decrease in N_2_ adsorption amount. These results indicate that the introduction of Ni does not create a new pore system. Instead, Ni species are more likely associated with defect sites or distributed within/near the accessible pore environment, leading to partial occupation of pore entrances or internal pore space.

The SEM images in Figure 1e–g show that the prepared samples maintain the characteristic octahedral morphology of NH_2_-UiO-66-derived materials. UN presents an octahedral morphology with an average particle size of approximately 300 nm, and Ni/UN still preserves this morphology after Ni loading. This observation agrees well with the XRD and FTIR results, collectively confirming that Ni modification does not induce obvious framework collapse or severe morphological destruction.

**Figure 2:**
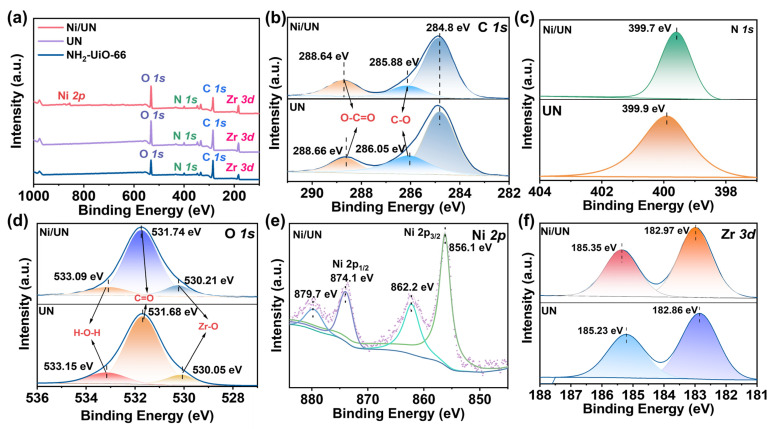
(**a**) XPS full spectra of NH_2_-UiO-66, UN and Ni/UN. (**b**) The C 1s, (**c**) N 1s, (**d**) O 1s, and (**f**) Zr 3d spectra of UN and Ni/UN, respectively. (**e**) The Ni 2p spectrum of Ni/UN.

As shown in Figure 2, the survey XPS spectra display the characteristic signals of C 1s, O 1s, N 1s and Zr 3d for the NH_2_-UiO-66-based samples, which is consistent with the elemental composition of amino-functionalized Zr-MOFs constructed from Zr-oxo clusters and aminoterephthalate linkers. The Ni 2p signal is only visible in the Ni-containing sample, directly indicating the successful introduction of Ni species into the sample. Similar XPS survey features of NH_2_-UiO-66, including C 1s, O 1s, N 1s and Zr 3d signals, have been reported in NH_2_-UiO-66-based photocatalytic systems. The high-resolution C 1s spectra can be deconvoluted into several carbon-related components. According to the labels in Figure 1b, the fitted peaks include O-C=O and C-O species, which correspond to oxygen-containing carbon environments from the carboxylate linker and related surface carbon species [32,33]. The presence of these components indicates that the organic linker-related carbon environments remain detectable after modification. The N 1s region shows a dominant N-containing signal for the samples, confirming that nitrogen species associated with the amino-functionalized framework are still present [22]. The O 1s spectra are fitted with multiple oxygen-related components. According to the labels in Figure 2d, these components include C=O, H-O-H and Zr-O contributions, indicating the coexistence of carboxylate oxygen, adsorbed water or hydroxyl-related oxygen, and Zr-O framework oxygen [22]. This result is consistent with the structural characteristics of NH_2_-UiO-66, whose Zr_6_O_4_(OH)_4_ clusters contain μ_3_-O and μ_3_-OH groups connected with carboxylate ligands. The Zr 3d spectra show a characteristic spin–orbit doublet, indicating the presence of Zr species in the samples. Reported NH_2_-UiO-66 XPS studies commonly assign the Zr 3d doublet to Zr species in Zr-O coordination environments within the Zr-oxo cluster. The high-resolution Ni 2p XPS spectrum of Ni/UN was further deconvoluted to clarify the oxidation state of Ni. The fitted peaks at 856.1 and 874.1 eV are assigned to Ni 2p3/2 and Ni 2p1/2 and the ratio of the areas of the two peaks is approximately 2:1, indicating that Ni exists in the material in the form of Ni^2+^ species, while the peaks at 862.2 and 879.7 eV correspond to the characteristic shake-up satellite peaks of Ni^2+^ [34,35]. These results confirm that Ni mainly exists as Ni^2+^ in the Ni/UN catalyst [36,37], and it may be more likely to coordinate with oxygen in metal clusters that lack part of Zr, forming a dual active center consisting of Ni and Zr [35,36,38].

**Figure 3:**
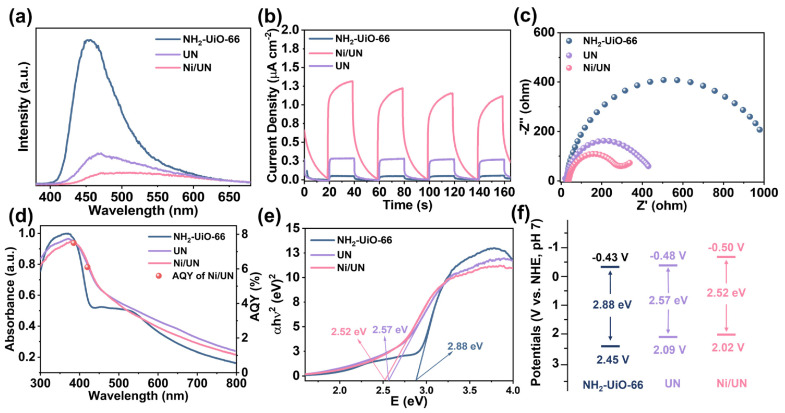
(**a**) Steady-state PL spectra, (**b**) transient photocurrent responses under chopped light irradiation, (**c**) EIS Nyquist plots, (**d**) solid ultraviolet absorption spectra and AQY of Ni/UN, (**e**) band gap, (**f**) band gap position diagrams calculated based on the Mott–Schottky curves and the optical band gap values of NH_2_-UiO-66, UN and Ni/UN, respectively.

As shown in Figure 3a, pristine NH_2_-UiO-66 exhibits the strongest steady-state PL emission among the three samples, while the emission intensity is markedly decreased after vacuum treatment and further reduced for Ni/UN. Since steady-state PL mainly reflects radiative recombination of photoinduced charge carriers, the lower PL intensity of UN and Ni/UN indicates that their radiative recombination signal is weakened relative to pristine NH_2_-UiO-66 [39]. The transient photocurrent responses in Figure 3b show reproducible current increases and decreases upon repeated light-on/off switching, confirming that all samples possess a detectable photoresponse under the test conditions. The photocurrent intensity indicates that Ni/UN generates the strongest photoinduced current response among the three samples [40]. In parallel, the EIS Nyquist plots in Figure 3c display the largest semicircle radius for NH_2_-UiO-66, a smaller radius for UN, and the smallest radius for Ni/UN. According to common photoelectrochemical analysis, a smaller EIS semicircle corresponds to lower interfacial charge-transfer resistance, while a higher photocurrent response is usually associated with more effective photoinduced charge separation and migration under identical testing conditions [41]. The critical role of Ni introduction is to regulate the local coordination and electronic structure of defect-engineered NH_2_-UiO-66, thereby facilitating the migration of photogenerated electrons and lowering the interfacial charge-transfer resistance, as evidenced by the decreased PL intensity, enhanced photocurrent response and reduced EIS semicircle radius of Ni/UN. In addition, the Ni-modified sample exhibits a narrower band gap and a slightly more negative conduction-band position, which further favors electron transfer toward O_2_ reduction and contributes to the improved H_2_O_2_ photosynthesis performance.

As shown in Figure 3d, the absorption edge of the material further redshifts after loading Ni. Not only that, the AQY values of the Ni/UN catalyst under the two wavelengths of 385 nm and 420 nm were 7.1% and 6.5%, respectively. The band gap width of the material was calculated based on the solid ultraviolet absorption spectrum and the Tauc equation, as shown in Figure 3e. The Mott–Schottky curves and Tauc results further reveal changes in the electronic structure of the samples. The conduction band positions of NH_2_-UiO-66, UN and Ni/UN were measured using the Mott–Schottky curves (Figures S1–S3). The valence band position can be calculated based on the conduction band position and the band gap width, as shown in Figure 3f. According to Figure 3f, the valence-band positions of NH_2_-UiO-66, UN and Ni/UN are estimated to be ~2.45, 2.09 and 2.02 V, respectively. The corresponding optical band gaps obtained from the Tauc plots are ~2.88 eV for NH_2_-UiO-66 and ~2.57 eV for UN, while ~2.52 eV for Ni/UN. Based on the band diagrams in Figure 3f, the conduction-band positions are −0.43, −0.48 and −0.50 V for NH_2_-UiO-66, UN and Ni/UN, respectively. These data directly show that, compared with pristine NH_2_-UiO-66, both UN and Ni/UN exhibit narrower band gaps, less positive valence-band positions and slightly more negative conduction-band positions [42], which are favorable for photocatalysis.

**Figure 4:**
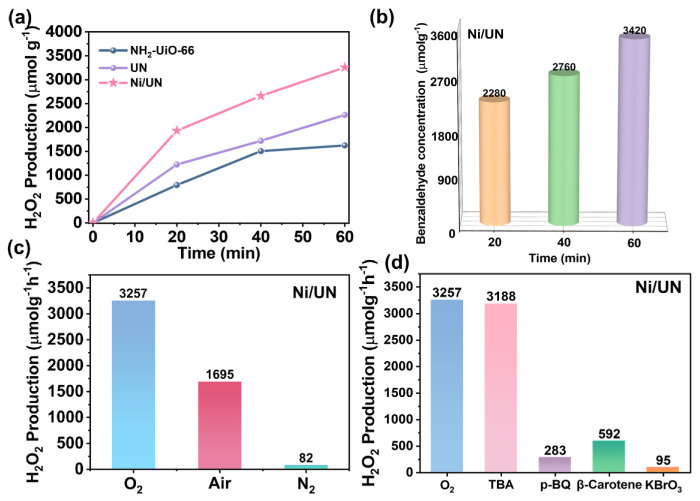
(**a**) Time-dependent H_2_O_2_ production over NH_2_-UiO-66, UN and Ni/UN. (**b**) Time-dependent benzaldehyde concentration, (**c**) effect of reaction atmosphere on H_2_O_2_ production, and (**d**) H_2_O_2_ production in the presence of different scavengers for Ni/UN, respectively.

As shown in Figure 4a, all tested samples exhibit continuously increased H_2_O_2_ production within 60 min, indicating that H_2_O_2_ accumulates progressively during the photocatalytic reaction period. The standard curve of hydrogen peroxide is shown in Figure S4. Through experiments, it was found that the photocatalytic H_2_O_2_ synthesis performance of the material was further enhanced after loading Ni. Moreover, by adjusting the loading concentration, it was discovered that when the loading concentration was high, the influence of Ni on Zr would increase, which was not conducive to the photocatalytic reaction. When the loading concentration was low, the synergistic effect between Ni and Zr could not reach its maximum. This result directly demonstrates that Ni introduction improves the H_2_O_2_ production performance of UN under the present experimental conditions. Figure 4b shows that the benzaldehyde concentration increases from 2280 μmol g^−1^ at 20 min to 2760 μmol g^−1^ at 40 min and further to 3420 μmol g^−1^ at 60 min. The concentration at 60 min is approximately 1.50 times that at 20 min, indicating continuous formation and accumulation of benzaldehyde during irradiation. The continuous formation of benzaldehyde also indicates that benzyl alcohol is not merely used as a reaction medium but directly participates in the oxidative half-reaction during photocatalysis. In the Ni/UN system, the benzaldehyde concentration increased from 2280 μmol g^−1^ at 20 min to 3420 μmol g^−1^ at 60 min, demonstrating that benzyl alcohol was continuously oxidized under light irradiation. This oxidation process can consume photogenerated holes and act as an electron-donating pathway, thereby suppressing charge recombination and leaving more photogenerated electrons available for the reduction in O_2_ toward H_2_O_2_ formation. This interpretation is also supported by the scavenger experiments, in which the electron scavenger KBrO_3_ sharply decreased the H_2_O_2_ production rate, while the O_2_-related scavengers p-BQ and β-carotene also caused significant inhibition. Therefore, the present data suggest that benzyl alcohol promotes H_2_O_2_ photosynthesis by coupling benzyl alcohol oxidation to benzaldehyde with the reductive O_2_-to-H_2_O_2_ pathway, rather than serving as an inert solvent [43,44]. The standard curve of benzaldehyde is shown in Figure S5. And no benzoic acid is produced. To rule out the possibility that H_2_O_2_ is produced spontaneously by benzaldehyde after its decomposition, we conducted a comparison experiment with and without a catalyst [45] (Figure S6). The experimental results showed that the contribution of H_2_O_2_ generated by benzaldehyde and benzyl alcohol in the absence of a catalyst to the total H_2_O_2_ in the system was extremely small, not exceeding 5% of the total. To exclude the possible homogeneous autophotocatalytic contribution from the benzyl alcohol/benzaldehyde system, we performed additional control experiments using 1-octanol as a non-aromatic alcohol electron donor. In contrast to benzaldehyde, the oxidation products of 1-octanol do not act as visible-light-active molecular photocatalysts. The control experiment showed that 1-octanol alone under LED irradiation produced negligible H_2_O_2_, whereas the Ni/UN catalyst still generated detectable H_2_O_2_ in the 1-octanol/O_2_ system. (Figure S7) These results indicate that the high H_2_O_2_ production in the benzyl alcohol system cannot be mainly attributed to a homogeneous alcohol/aldehyde autophotocatalytic pathway, but is predominantly associated with the Ni/UN-catalyzed photoinduced O_2_ reduction process. In addition, a cyclic test was conducted on Ni/UN, and it was found that the photocatalytic performance of the catalyst decreased by less than 5% after seven cycles, indicating that the catalyst has good cyclic stability [45] (Figure S8).

The atmosphere-dependent experiments in Figure 4c further show that Ni/UN delivers the highest H_2_O_2_ production rate under an O_2_ atmosphere, reaching 3257 μmol g^−1^ h^−1^. When the atmospheric conditions changed to air and N_2_, the photocatalytic performance of the catalyst gradually decreased, indicating that O_2_ plays an important role in the photocatalytic synthesis of H_2_O_2_ by the catalyst. These data directly indicate that the H_2_O_2_ production performance is strongly dependent on the reaction atmosphere, and an O_2_-rich environment is more favorable for H_2_O_2_ generation than air or N_2_ [46]. This trend is consistent with the general understanding that photocatalytic H_2_O_2_ synthesis commonly involves oxygen reduction and/or water oxidation processes [47]. The scavenger experiments in Figure 4d provide further evidence for the reaction-related active species. Under O_2_ without additional scavenger, Ni/UN produces 3257 μmol g^−1^ h^−1^ H_2_O_2_. After adding TBA, the production rate remains at 3188 μmol g^−1^ h^−1^, corresponding to only a 2.1% decrease, suggesting that ·OH-related processes are not significantly associated with the observed H_2_O_2_ generation under these conditions [48]. In contrast, the addition of p-BQ, β-carotene and KBrO_3_ sharply decreases the H_2_O_2_ production rate to 283, 592 and 95 μmol g^−1^ h^−1^, corresponding to inhibition degrees of approximately 91.3%, 81.8% and 97.1%, respectively. According to the commonly used assignments in photocatalytic scavenger tests, p-BQ is often used to probe ·O_2_^−^, β-carotene is used for ^1^O_2_ quenching, and KBrO_3_ acts as an electron scavenger [49]. Therefore, the strong inhibition caused by these additives indicates that photogenerated electrons and oxygen-derived reactive species are closely related to H_2_O_2_ formation in this system [50].

**Figure 5:**
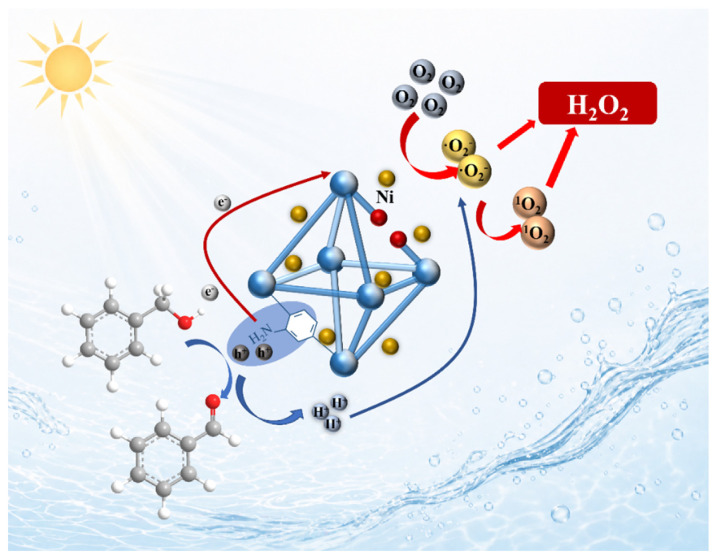
Possible photocatalytic H_2_O_2_ generation pathway over Ni/UN under light irradiation.

Figure 5 schematically illustrates the proposed photocatalytic H_2_O_2_ generation pathway over a Ni/UN photocatalyst. Upon light irradiation, photogenerated electrons and holes are generated and spatially directed toward the reduction and oxidation processes, respectively. The reduction pathway depicted in the scheme involves O_2_ activation followed by the formation of ·O_2_^−^, suggesting that an indirect stepwise oxygen reduction route contributes to H_2_O_2_ production. In addition, ^1^O_2_ is also shown as a reactive oxygen species associated with the final formation of H_2_O_2_, indicating the possible participation of multiple oxygen-derived intermediates. At the photogenerated hole end, benzyl alcohol is converted into benzaldehyde. The photogenerated charge carriers participate in the redox to produce H_2_O_2_ and benzaldehyde simultaneously.

## 3. Conclusions

In summary, Ni-modified defect-engineered NH_2_-UiO-66 photocatalysts were successfully constructed by introducing Ni species into the vacuum-treated UN. Structural characterizations confirmed that the UiO-type crystalline framework, characteristic functional groups and octahedral morphology were well retained after Ni loading, while XPS analysis verified the successful incorporation of Ni species mainly in the divalent state. Compared with pristine NH_2_-UiO-66 and UN, Ni/UN showed more efficient photoinduced charge separation and transfer, as evidenced by the weakened PL emission, enhanced transient photocurrent response and reduced interfacial charge-transfer resistance. In addition, Ni/UN exhibited a narrowed band gap of approximately 2.52 eV and a slightly more negative conduction-band position of −0.50 V, indicating that Ni modification and defect engineering jointly optimized the electronic structure of NH_2_-UiO-66.

Photocatalytic H_2_O_2_ production is considered a promising route for sustainable and decentralized H_2_O_2_ generation because it can convert light energy into chemical oxidants under mild conditions while avoiding the transportation and storage issues associated with concentrated H_2_O_2_. In this regard, the Ni/UN photocatalytic system developed in this work may provide a potential platform for on-site H_2_O_2_ generation, especially considering that photocatalytically produced H_2_O_2_ has been discussed for environmental remediation, disinfection, and the production of high-value chemicals. Moreover, the present system couples H_2_O_2_ photosynthesis with the selective oxidation of benzyl alcohol to benzaldehyde, indicating its potential relevance to green oxidation processes in which the oxidative and reductive half-reactions are simultaneously utilized to generate value-added products. Although further studies under continuous-flow operation, real wastewater conditions, and broader substrate scopes are still required, this work provides a feasible strategy for constructing MOF-based photocatalysts through Ni coordination regulation and defect-induced charge-transfer optimization for coupled H_2_O_2_ photosynthesis and selective alcohol oxidation.

## Data Availability

The original contributions presented in this study are included in the article/Supplementary Material. Further inquiries can be directed to the corresponding authors.

## Supplementary Materials

The following supporting information can be downloaded at https://www.mdpi.com/article/10.3390/nano16100626/s1: Figure S1. The Mott-Schottky curves of NH2-UiO-66. Figure S2. The Mott-Schottky curves of UN. Figure S3. The Mott-Schottky curves of Ni/UN. Figure S4. Hydrogen peroxide calibration curve. Figure S5. Benzaldehyde calibration curve. Figure S6. Photocatalytic H_2_O_2_ evolution rate with and without catalyst. Figure S7. Comparison experiments of photocatalytic H_2_O_2_ evolution between BA and 1-octanol. Figure S8. Cyclic tests of Ni/UN. Figure S9. O_2_ saturation tests of Ni/UN.
