# Supplementary material for: Ni-Modified Defect-Engineered NH2-UiO-66 for Efficient H2O2 Photosynthesis Coupled with Benzyl Alcohol Oxidation
Source: Nanomaterials (Basel). 2026 May 19;16(10):626. doi: 10.3390/nano16100626 (PMC13209578; doi:10.3390/nano16100626)
Supplement: Supplementary file 1 [file nanomaterials-16-00626-s001.zip › nanomaterials-4327943-supplementary.pdf]

Supporting Information

Experimental Section

Materials and reagents

All raw materials were purchased directly from the reagent company without further purification. Tetraclorozirconium ($ZrCl_4$, ≥99.7%), acetic acid ($C_2H_4O_2$, ≥99.7%), 2-aminoxydiphenylformic acid ($C_8H_6O_4$, 99%) were purchased from Aladdin. The N,N-dimethylformamide (C_3H_3NO, AR), and anhydrous methanol (C_2H_5OH, AR) were purchased from China Pharmaceutical Group Chemical Reagent Co., Ltd.

Synthesis of NH_2-UiO-66

46.6 mg of $ZrCl_4$ and 36.2 mg of 2-amino-4-nitrophenylacetic acid were separately weighed and dissolved in 20 mL of DMF. Ultrasonic treatment was applied to ensure complete dissolution of the solutes. Then, 2.3 mL of acetic acid was added to the transparent solution, followed by transferring the mixed solution to a 50 mL stainless steel high-pressure reactor with a polytetrafluoroethylene inner lining. The high-pressure reactor was placed in a drying oven and heated at 120 °C for 24 h. After the reaction, the product was cooled at a rate of 5 °C/min to room temperature, centrifuged for collection, and washed three times with DMF and methanol respectively. Then, it was dried at 70 °C in a vacuum oven for 12 h to obtain the octahedral NH_2-UiO-66.

Preparation of defective NH_2-UiO-66

The synthesized NH_2-UiO-66 was placed in a porcelain boat, then put into a vacuum oven and kept at a temperature of 200 °C to obtain defective NH_2-UiO-66, which was denoted as UN.

Preparation of Ni/UN

Add 200 mg of UN to 15 mL of methanol, then add $NiCl_2·6H_2O$ of 24 mg (3% of UN quality) and stir for 2 h. Centrifuge and collect the product, and wash it three times

with methanol to remove unreacted metal ions. Then, the solid was dispersed in 10 mL of methanol. Subsequently, 200 μL of hydrazine hydrate was added under vigorous stirring for 30 min. After that, the product was separated by centrifugation, washed with methanol three times, and dried at room temperature under vacuum to obtain the product.

Characterizations

The chemical structure of the polymers was characterized by the ATR attachment of Fourier transform infrared (FT-IR) spectra (Nicolet spectrometer). The crystal structure was investigated by powder X-ray diffraction (PXRD, Bruker D8 advance) using Cu Kα radiation. Microscopic morphology was observed by scanning electron microscope (SEM, Hitachi SU8010). The Brunauer-Emmett-Teller (BET) surface area and pore volumes of the polymers were analyzed by nitrogen adsorption-desorption isotherms on Micrometrics ASAP 2040. The spectral response range of polymers were evaluated by UV-Vis diffuse reflectance spectra (DRS, Shimadzu UV-3100).

Benzaldehyde (BAD) Detection

0.3 mL organic phase in reaction system was firstly filtered using a syringe with a 0.22 μm micron nylon filter to remove the photocatalyst. Then the clear organic solution was feed into the gas chromatography sample bottle to detect. The testing conditions for gas chromatography were set as follows: sample injection volume 0.5 μL, 50 °C maintenance for 2 minutes, temperature from 50 °C to 190 °C with a heating rate 10 °C min^{-1}, column type P / N.AA-003-08407 30 m * 0.25 mm * 0.25 μm. The final amount of BA oxidation products were estimated according to the standard curve of BAD or Benzoic acid content along with chromatographic peak area.

AQY tests

We conducted AQY tests on Ni/UN at wavelengths of 385 nm and 420 nm respectively. The test results are shown in the following figure. The AQY values of this catalyst at wavelengths of 385 nm and 420 nm are 7.1% and 6.5% respectively.

The apparent quantum yield (AQY) measurement for hydrogen peroxide was measured with monochromatic light obtained by using band pass filters of 385 nm and 420 nm. The irradiation area was controlled as 2.56×3.14 cm2. AQY for H2O2 production at monochromatic light irradiation was estimated as below equation.

$$\mathrm{AQY} = \frac{2 \times \text{Number of produced } \mathrm{H_2O_2} \text{ molecules}}{\text{Number of incident photons}} = \frac{2 \times \mathrm{M} \times \mathrm{N_A}}{\mathrm{S} \times \mathrm{P} \times \mathrm{t} \times \frac{\lambda}{\mathrm{h} \times \mathrm{c}}} \times 100\%$$

Where, M is the amount of hydrogen peroxide molecules (mol), NA is Avogadro constant (6.022 × 1023 /mol), h is the Planck constant (6.626 × 10-34 J·s), c is the speed of light (3 × 108 m/s), S is the irradiation area (cm2), P is the intensity of irradiation light (W/cm2), t is the photoreaction time (s), λ is the wavelength of the monochromatic light (m).

Electrochemical measurements

The electrochemical workstation used an Ag/AgCl electrode, platinum wire, and catalyst as the three-electrode system. 0.2 M sodium sulfate and 10 mM potassium ferrocyanide/ferrocyanide potassium mixed solution were used as electrolytes. NH_2-UiO-66, UN, and Ni/UN were used as photocatalytic electrodes. The preparation method of the photocatalytic electrode was as follows: 3 mg of NH_2-UiO-66, or UN, or Ni/UN were dispersed in 1 mL of DMF and 50 μL of naphthol mixed solution, and then ultrasonicated to achieve uniform dispersion. Subsequently, 10 μL of the above suspension was dropped onto a 0.25 cm^2 FTO glass using a pipette. After the sample dried naturally, the photocatalytic and photoelectrochemical performance test was conducted.

Photocatalytic performance tests

5 mg of photocatalyst and 20 mL of Benzoic alcohol were added to the container. Ultrasound was used to evenly disperse the catalyst. Following ultrasonic dispersion of the catalyst, a multi-channel reactor (PCX-50C Multi-Channel Photochemical Reaction System, Beijing Perfectlight, China, light intensity: 83 mW cm^{-2}) was employed for light irradiation. Then, a balloon filled with high-purity oxygen was used to bubble the uniformly dispersed photocatalyst suspension for 20 min. All the

air in the reaction system was removed to ensure the pure oxygen conditions required for the test. The light source used in the photocatalytic reaction was an LED lamp. The reaction system used circulating cold water to conduct the photocatalytic reaction at 25 °C. Samples were taken every 20 min. The H_2O_2 content was detected using the colorimetric method with a UV spectrophotometer.

The colorimetric method uses N, N-diethyl-p-phenylenediamine sulfate (DPD) to determine the concentration of hydrogen peroxide (H_2O_2). A phosphate sodium buffer solution (pH=6) needs to be prepared before the test.

Preparation of phosphate sodium buffer solution (pH=6): Using volumetric flasks, prepare 0.5 mol/L aqueous solutions of sodium dihydrogen phosphate anhydrous and sodium dihydrogen phosphate monohydrate, respectively. Gradually add the anhydrous sodium dihydrogen phosphate solution into the monohydrate counterpart to adjust the pH value. Allow the pH meter reading to stabilize at 6.0, and perform the preparation in triplicate to confirm a stable pH of 6.0 for all replicates.

Preparation of DPD solution: Accurately weigh 0.1 g of N, N-diethyl-p-phenylenediamine sulfate, and dissolve it in 10 mL of 0.05 M sulfuric acid (H_2SO_4). Subject the mixture to ultrasonication until the solute is completely dissolved to obtain the DPD stock solution.

Preparation of POD solution: Accurately weigh 5 mg of peroxidase and dissolve it in 5 mL of deionized water. Perform ultrasonication to completely dissolve the enzyme, yielding the POD solution.

Preparation of reference solution: Accurately pipette 3 mL of sodium phosphate buffer solution (pH = 6.0) into a 10 mL colorimetric tube. Then, sequentially add 6.9 mL of deionized water, 0.05 mL of DPD solution, and 0.05 mL of POD solution. Shake the tube thoroughly to ensure uniform mixing, and this mixture is defined as the reference solution.

H_2O_2 detection methods

After 20 min of light exposure, accurately pipette 3 mL of sodium phosphate buffer solution (pH = 6.0) into 10 mL colorimetric tubes. Next, use a 200 μL disposable

syringe to withdraw 1 mL of sample solution from the reactor, filter the catalyst through a 0.22 μm PES filter membrane, and add the clarified sample solution to the aforementioned colorimetric tubes containing the sodium phosphate buffer solution. Subsequently, add 0.05 mL of DPD solution, 0.05 mL of POD solution, and 6.7 mL of deionized water in sequence, and shake thoroughly to ensure uniform mixing. A UV-visible spectrophotometer (UV-9000s, Metash) was employed to determine the absorbance of the above mixed solution in the wavelength range of 400 nm – 600 nm, and the absorbance value at a wavelength of 552 nm was recorded. For the calibration curve construction, the 30% H_2O_2 stock solution was diluted to a concentration range of 2–35 μM, and the absorbance at each corresponding concentration was measured simultaneously. A linear relationship plot between H_2O_2 concentration and the corresponding absorbance intensity was then drawn, which served as the standard curve (Figure S1).

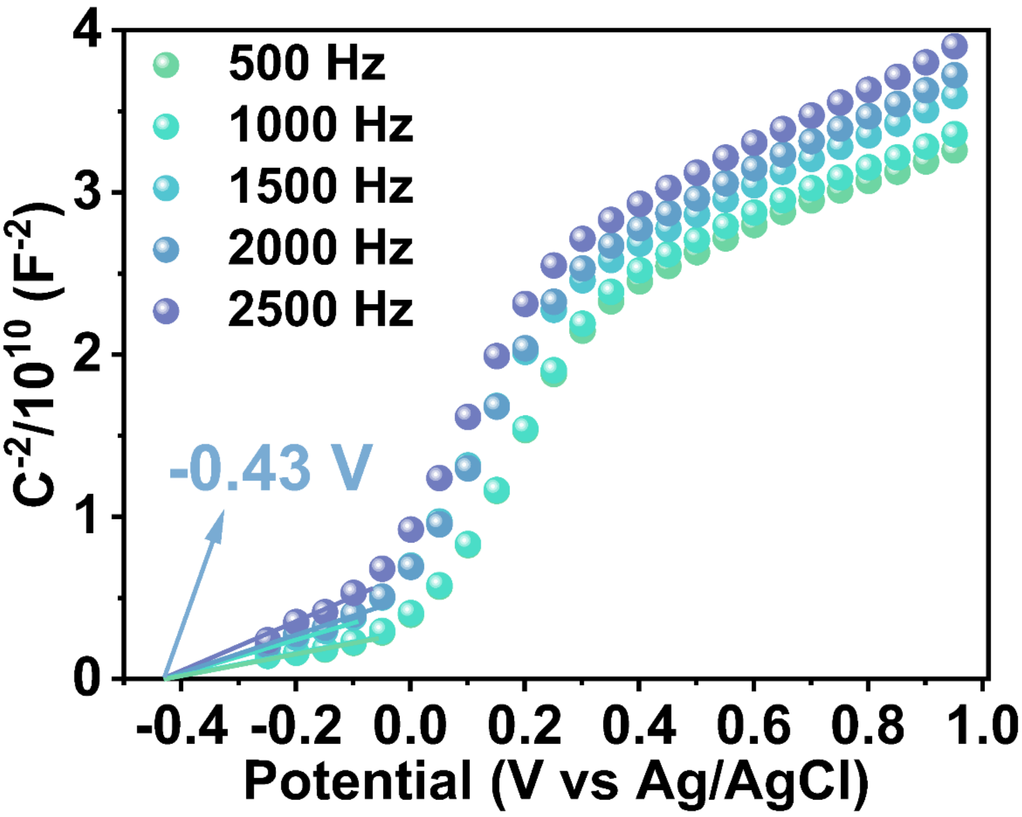

Supplementary Figure S1. The Mott-Schottky curves of NH_2-UiO-66.

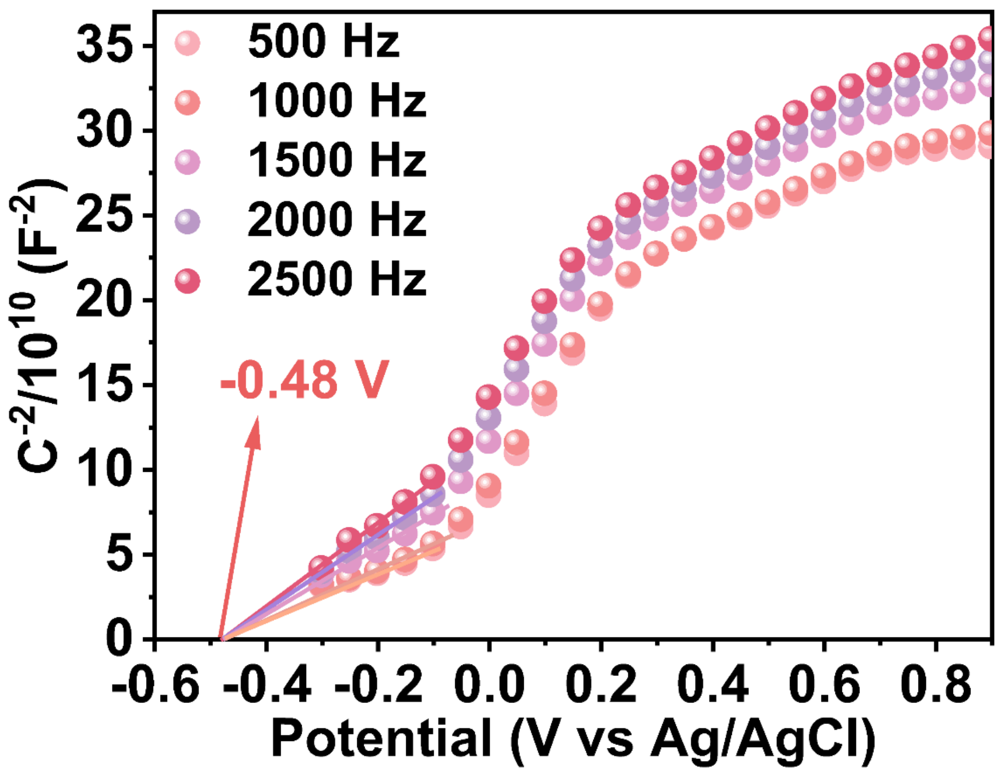

Supplementary Figure S2. The Mott-Schottky curves of UN.

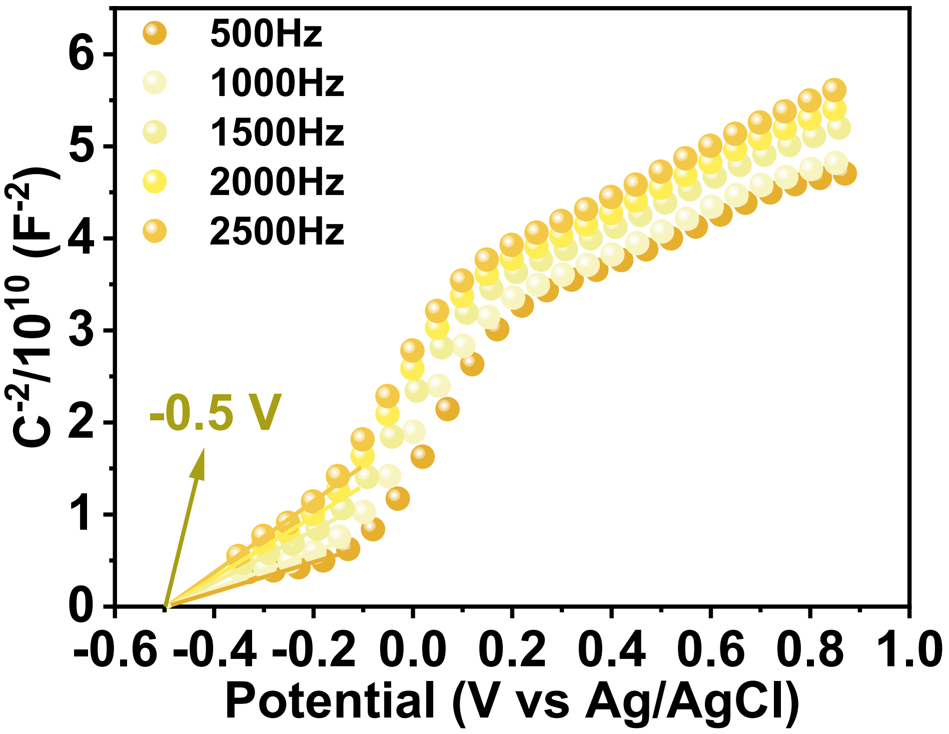

Supplementary Figure S3. The Mott-Schottky curves of Ni/UN.

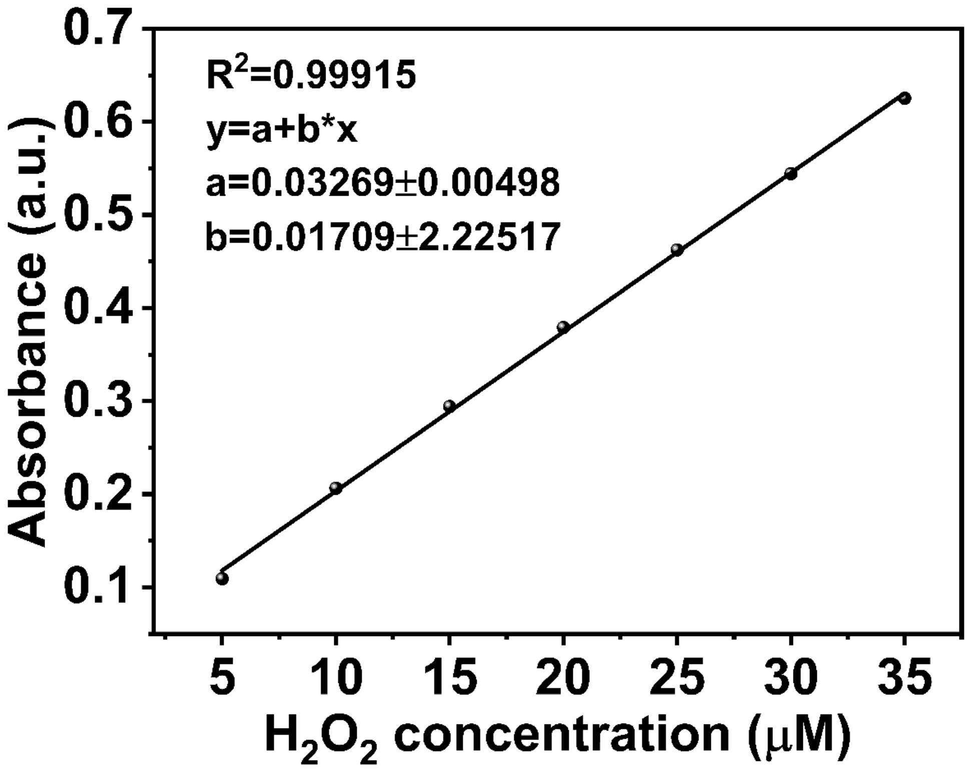

Supplementary Figure S4. Hydrogen peroxide calibration curve.

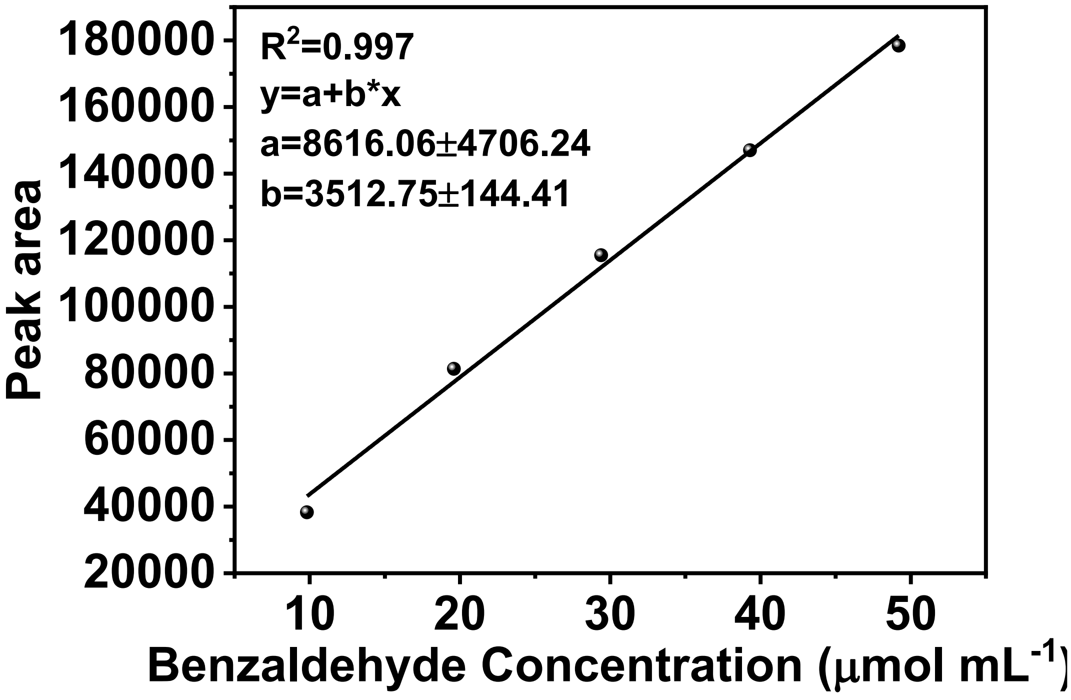

Supplementary Figure S5. Benzaldehyde calibration curve.

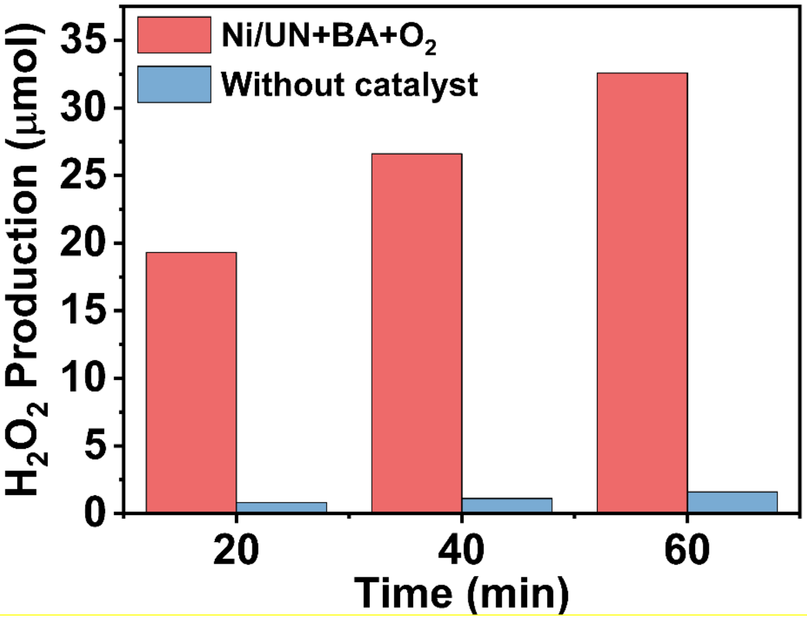

Supplementary Figure S6. Photocatalytic H_2O_2 evolution rate with and without catalyst.

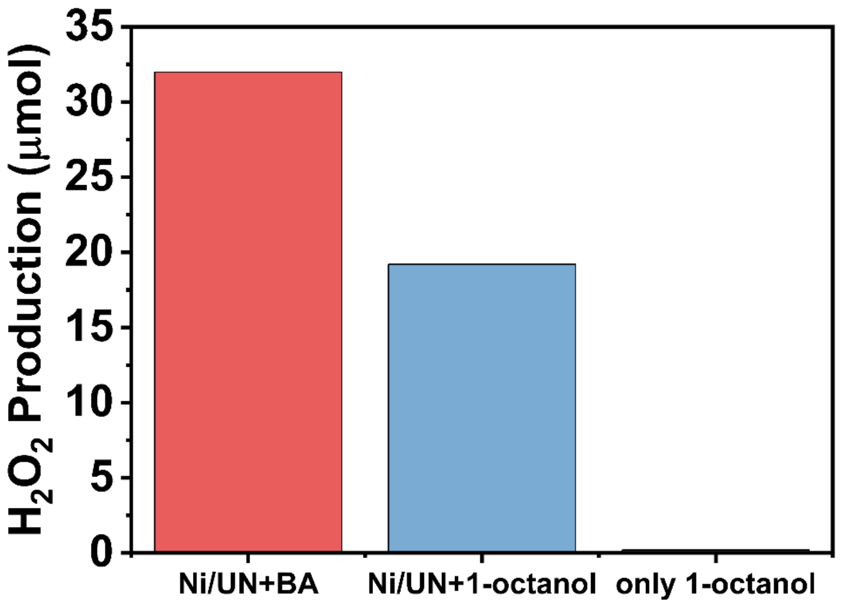

Supplementary Figure S7. Comparison experiments of photocatalytic H_2O_2 evolution between BA and 1-octanol.

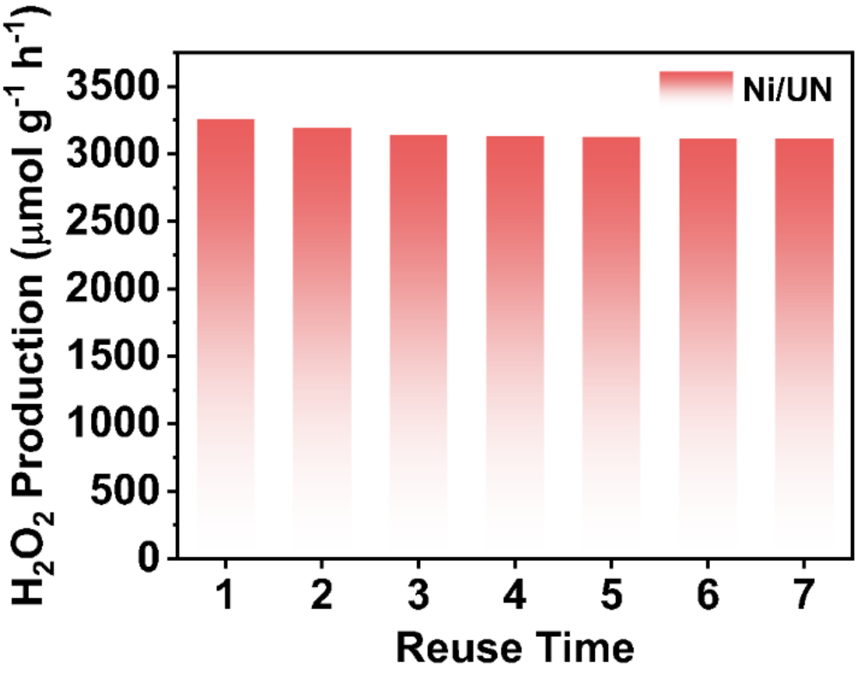

Supplementary Figure S8. Cyclic tests of Ni/UN.

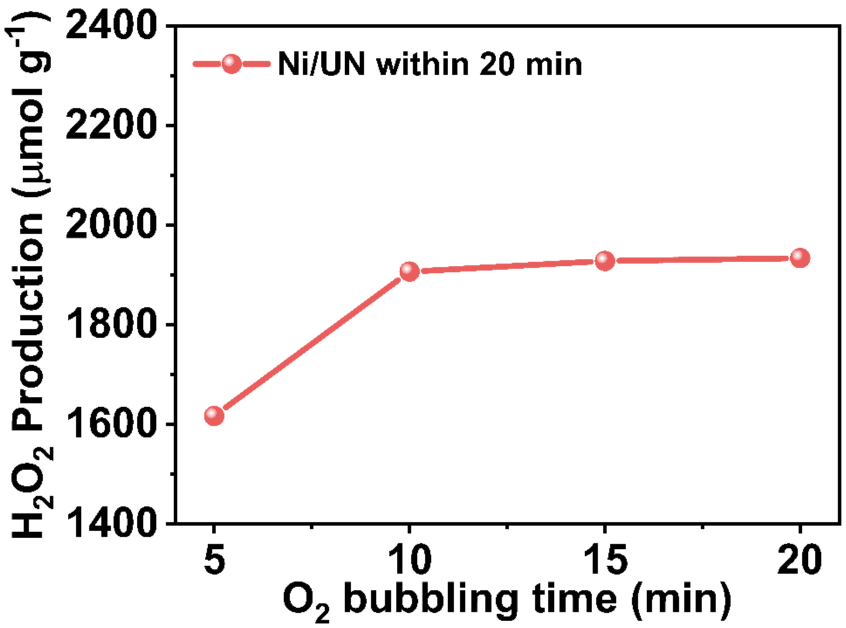

Supplementary Figure S9. O_2 saturation tests of Ni/UN.
